# Cystoid Macular Edema Following Phacoemulsification and Posterior Chamber Intraocular Lens Surgery: An Aspect From the Past to the Present

**DOI:** 10.7759/cureus.89358

**Published:** 2025-08-04

**Authors:** Sevgi Tongal, Ali Osman Saatci, Mehmet Ergin

**Affiliations:** 1 Department of Ophthalmology, Beyoğlu Ophthalmic Education and Research Hospital, Istanbul, TUR; 2 Department of Ophthalmology, Faculty of Medicine, Dokuz Eylul University, Izmir, TUR

**Keywords:** cystoid macular edema, fundus fluorescein angiography, optical coherence tomography, phacoemulsification, posterior chamber intraocular lens

## Abstract

Background

Cystoid macular edema (CME) is a known cause of vision loss after cataract surgery. This study examines its incidence following phacoemulsification and intraocular lens implantation (1997-1998) and discusses how evolving surgical and diagnostic techniques may have influenced its occurrence.

Methods

Between September 1997 and June 1998, 54 eyes of 51 patients who underwent uneventful cataract surgery were included in the study. Best-corrected visual acuity (BCVA) was assessed, and fundus fluorescein angiography (FFA) was performed at postoperative weeks 6, 12, and 24 to evaluate CME according to the Yanuzzi classification.

Results

At week 6, angiographic CME was observed in six of 54 eyes (11.1%). At month 3, CME was present in three of 36 eyes (8.3%), and by month 6, it persisted in three of 13 eyes (23.1%). Among the initial CME cases, three resolved by the third month. Notably, five of six CME cases (15.1%) occurred in patients aged ≥60, while only one case (4.7%) was seen in those under 60. None progressed to clinically significant CME.

Conclusions

CME can still occur after uncomplicated cataract surgery, though typically mild and transient. Advances such as optical coherence tomography have enhanced early detection and management, reinforcing the need for vigilant postoperative monitoring.

## Introduction

Cystoid macular edema (CME) is a well-known complication following cataract surgery, characterized by fluid accumulation in the outer plexiform layer of the retina due to a breakdown of the blood-retina barrier. Although advancements in surgical techniques, including phacoemulsification and posterior chamber intraocular lens (IOL) implantation, have significantly improved visual outcomes, CME remains a concern due to its potential to cause retinal thickening and impaired vision. While many cases resolve spontaneously, some may persist and require medical intervention to prevent long-term visual impairment [1].

Several factors contribute to the development of CME, including intraoperative complications, pre-existing ocular pathologies, and systemic conditions such as diabetes mellitus and vascular diseases. Additionally, patient age appears to play a role, with older individuals exhibiting a higher prevalence of CME, possibly due to age-related vascular changes and inflammatory responses [1].

CME can present in different forms depending on its severity and clinical impact. Angiographic CME is the most common type and is often asymptomatic, detected through fundus fluorescein angiography (FFA), which reveals perifoveal capillary leakage. Clinically significant CME is associated with a measurable decline in visual acuity, affecting daily activities and overall quality of life. Chronic CME, persisting for more than six months, poses a greater risk of irreversible retinal damage and long-term visual deficits [2]. The severity of CME is assessed using various imaging techniques, including optical coherence tomography (OCT) and FFA, which help evaluate retinal thickening and vascular leakage patterns [3,4].

Notably, at the time this study was conducted, phacoemulsification was still in its early stages of adoption, and foldable IOLs had only recently become available. Although the surgical approach was intended to be sutureless, larger incisions were often necessary to accommodate the implantation of available IOLs, which were chosen based on availability rather than standardized criteria. Technological and logistical limitations of that period also influenced surgical practices, including IOL selection and incision techniques [5].

FFA was the primary modality for detecting CME, as OCT was not yet widely accessible in routine clinical use. Today, OCT has largely replaced FFA for CME assessment due to its noninvasive nature, rapid imaging capability, and higher spatial resolution. Therefore, this study also provides a valuable historical perspective on CME evaluation during the early phase of modern cataract surgery.

This study aims to evaluate the incidence of CME following phacoemulsification and posterior chamber IOL implantation, specifically in cases without intraoperative complications or predisposing systemic and ocular conditions. By analyzing postoperative CME occurrence in this setting, the research seeks to provide a clearer understanding of its frequency, natural course, and potential risk factors in an otherwise uncomplicated surgical population.

## Materials and methods

Study design and setting

This prospective observational study was conducted at the Department of Ophthalmology, Dokuz Eylul University Faculty of Medicine, Izmir, Türkiye, between September 1997 and June 1998 to evaluate the incidence of CME following phacoemulsification and posterior chamber IOL implantation.

Ethical considerations

This study was conducted in 1998 at the Department of Ophthalmology, Dokuz Eylul University Faculty of Medicine, as part of a residency thesis (Thesis No: 70816) registered in the National Thesis Center of the Council of Higher Education. At the time of the study, obtaining approval from an ethics committee was not legally required for retrospective and observational studies conducted as part of educational training. All patients were included after providing informed verbal consent, which included a discussion of the potential risks associated with fundus fluorescein angiography, and the study was conducted in accordance with the principles of the Declaration of Helsinki.

Patient selection

A total of 54 eyes from 51 patients, aged 37-78 years (mean=62.01 years), who underwent cataract surgery with phacoemulsification and posterior chamber IOL implantation were included. Patients were selected based on specific inclusion and exclusion criteria. Those with previous intraocular surgery, intraoperative complications such as posterior capsule rupture or vitreous loss, systemic conditions like diabetes mellitus, vasculitis, and collagen tissue diseases, or ocular pathologies such as uveitis, retinal detachment, glaucoma, and age-related macular degeneration were excluded. Additionally, patients undergoing combined surgical procedures were not included in the study.

Surgical procedure

All surgeries were performed under retrobulbar anesthesia, with midriasis achieved using 1% cyclopentolate, 10% phenylephrine, and 0.5% tropicamide. Retrobulbar anesthesia was administered using prilocaine 2% and bupivacaine 0.5%, followed by ocular massage to reduce intraocular pressure. The surgical procedure involved a corneal, limbal, or scleral tunnel incision of 2.8 mm, followed by continuous curvilinear capsulorhexis. Nucleus emulsification was performed using the divide-and-conquer technique. Depending on the IOL type, incisions were extended: acrylic and silicone foldable lenses were implanted through a 3.5-4-mm incision, while monoblock polymethylmethacrylate (PMMA) lenses required a 5-6-mm incision. Acrylic lenses in 21 eyes, silicone foldable lenses in 16 eyes, and PMMA lenses in 17 eyes were placed in the capsule. Single or cross sutures were placed. No sutures were placed in the eyes with foldable lenses. The procedure was completed with a subconjunctival injection of gentamicin and dexamethasone.

Postoperative care and follow-up

Postoperatively, patients were prescribed ofloxacin and prednisolone acetate eye drops. Ofloxacin was discontinued after one week, while prednisolone was tapered over four weeks. Follow-up examinations were conducted at postoperative week 6, month 3, and month 6, during which best-corrected visual acuity (BCVA) was assessed. FFA was performed using intravenous 20% sodium fluorescein (3 cc) to evaluate CME. The severity of CME was classified according to the Yannuzzi classification [2,6], which categorized leakage into five stages: Stage 0 (no leakage), Stage 1 (minimal perifoveal leakage), Stage 2 (mild perifoveal edema), Stage 3 (moderate perifoveal edema), and Stage 4 (severe and extensive perifoveal edema).

## Results

A total of 54 eyes from 51 patients, ranging in age from 37 to 78 years, with a mean age of 62.01 years, were evaluated according to the established criteria. Of the patients, 21 (21 eyes) were under 60 years of age, while 30 (33 eyes) were 60 years of age or older. Of the total, 29 were right eyes and 25 were left eyes. The sample included 30 males (32 eyes) and 21 females (22 eyes). Hypertension was detected in 10 eyes, and degenerative myopia was detected in two eyes. No other systemic or ocular diseases were detected. The development of CME was not observed in patients with hypertension and degenerative myopia.

**Figure 1:**
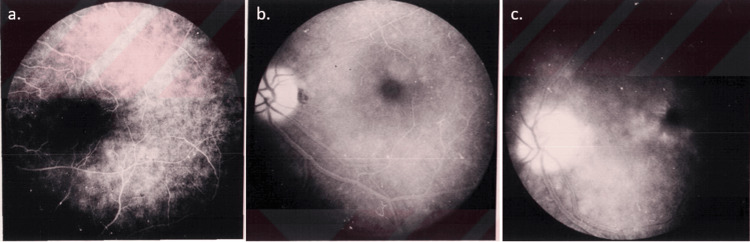
Stage 1 angiographic CME (a), Stage 2 angiographic CME (b), Stage 3 angiographic CME (c)

Early postoperative examinations were conducted, and the initial FFA was performed at the sixth week. The results were then evaluated according to the Yannuzzi classification. Angiographic CME was detected in six of 54 eyes (11.11%). Of these cases, four were classified as stage 1, one as stage 2, and one as stage 3 (Figure 1). None of the patients with CME exhibited a decline in visual acuity.

In the study population, one (4.7%) of 21 eyes under 60 years of age and five (15.1%) of 33 eyes aged 60 years and older exhibited angiographic CME (Table 1).

**Table 1:** Angiographic cystoid macular edema (CME) according to age

Age	Total number of eyes	Angiographic CME number (%)
Under 60 years old	21	1 (4.7%)
Over 60 years old	33	5 (15.1%)

Of the 54 eyes that underwent angiography at week six, 36 eyes were admitted to our clinic and underwent FFA at three months. Of the six eyes with angiographic CME at week six, all were re-evaluated at month three. In three of these eyes - one case with stage 2 and two cases with stage 1 CME - CME had resolved, while the remaining three (8.33%) showed persistent CME at the same severity level. None of the patients without angiographic CME at week six had CME at three months. The incidence of angiographic CME according to the time of angiography is shown in Table 2.

**Table 2:** Angiographic CME according to FFA performing time CME: cystoid macular edema; FFA: fundus fluorescein angiography

FFA performing time	Angiographic CME			
	Present	Absent		Total
6^th^ Week	6 (11.11%)	48		54
3^rd ^Month	3 (8.33%)	33		36
6^th^ Month	3 (24.07%)	10		13

At the six-month follow-up, FFA was performed on 13 eyes from 13 patients. Among these, the three eyes that had exhibited CME at the three-month visit continued to show angiographic CME at a similar severity level, with no deterioration in visual acuity. No new cases of CME were detected at month six (Table 3).

**Table 3:** Angiographic CME stage according to FFA performing time CME: cystoid macular edema; FFA: fundus fluorescein angiography

FFA Performing Time	Stage 0	Stage 1	Stage 2	Stage 3	Stage 4
6^th^ Week	48	4	1	1	0
3^rd ^Month	33	2	0	1	0
6^th^ Month	10	2	0	1	0

## Discussion

Phacoemulsification has revolutionized cataract surgery by allowing for a minimally invasive approach that preserves the anatomy and physiology of the eye as much as possible. This technique involves entering the eye through a small incision, performing a precise capsular incision, maintaining the integrity of the posterior capsule, and implanting the IOL through the same incision [5]. Despite advancements in surgical techniques, CME remains a recognized postoperative complication [7-9].

In our cohort, the incidence of angiographic CME at six weeks was 11.11%, which is comparable to similar studies using FFA during the same era. For example, Mangla et al. conducted an FFA-based study in a larger population with longer follow-up and reported similar trends [10]. In contrast, more recent large-scale registry analyses, such as those utilizing the IRIS® Registry (American Academy of Ophthalmology, San Francisco, USA), report CME rates as low as 0.8% at 90 days post-surgery - likely reflecting both improvements in surgical technique and the increased use of OCT for earlier and more sensitive detection [1].

Our study is of particular interest as it was conducted during the transitional period when phacoemulsification and foldable IOLs were first being introduced. At that time, sutureless techniques were emerging, but larger incisions were still common to accommodate rigid or early-generation foldable IOLs, and lens selection was largely dictated by availability. The exclusive use of FFA for CME detection in this study reflects the technological limitations of the era; OCT was not yet in widespread use. Today, OCT has largely replaced FFA due to its non-invasive nature, ease of use, and superior resolution, offering rapid and reproducible imaging for CME diagnosis and monitoring [11]. Studies incorporating both FFA and OCT have demonstrated good correlation, although OCT may detect CME earlier and in finer detail [12,13]. The transition from FFA to OCT not only represents a technological leap but also reflects broader trends in clinical practice favoring less invasive, more patient-friendly diagnostics.

In addition to improvements in IOL materials and imaging technologies, the evolution of phacoemulsification devices themselves has played a critical role in reducing postoperative complications such as CME. During the late 1990s, phaco systems typically relied on longitudinal ultrasound energy and peristaltic pump systems with manual fluidics, requiring higher energy and often resulting in suboptimal anterior chamber stability. In contrast, modern systems incorporate torsional or transversal ultrasound delivery and intelligent fluidics platforms - such as Active Fluidics™ (Alcon Pofessionals, Vernier, Switzerland) - which provide real-time pressure modulation and improved chamber control. These innovations reduce mechanical and thermal stress on intraocular tissues, thereby minimizing postoperative inflammation, one of the key contributors to CME [14,15]. The cumulative impact of these advances has not only improved surgical efficiency but also contributed to the decreased incidence and severity of CME in contemporary cataract surgery.

Our study highlights the correlation between age and CME incidence, with older patients demonstrating a higher prevalence (15.1% in patients aged 60 and above vs. 4.7% in younger patients). This aligns with previous research suggesting that age-related vascular changes and an increased inflammatory response contribute to blood-retina barrier disruption, predisposing elderly individuals to CME [2,3,16]. A systematic review by Ferro Desideri and colleagues confirmed that aging is a critical risk factor, further supporting the observed trend in our cohort [4].

Notably, while our study found a higher incidence of CME in patients aged 60 and above, recent large-scale registry analyses, such as the IRIS Registry, have reported increased CME rates in younger patients (<65 years) [1]. This discrepancy may reflect differences in patient selection, as our study excluded individuals with systemic or ocular risk factors and included only uncomplicated cases. Additionally, methodological differences - such as reliance on FFA in our study versus OCT-based detection in IRIS - may also influence reported incidence patterns.

Interestingly, while our study did not identify cases of clinically significant CME, the persistence of angiographic CME in three eyes at the six-month follow-up underscores the potential for long-term retinal alterations even in asymptomatic patients. This observation echoes findings emphasizing the roles of imaging modalities such as OCT in detecting subclinical CME, which may still impact retinal function over time [6,17,18].

The presence of preexisting ocular conditions significantly influences the development of CME following cataract surgery. For instance, patients with a history of epiretinal membrane (ERM) exhibit a higher likelihood of developing postoperative CME [19]. A study reported that eyes with preexisting ERM had a 7% incidence of postoperative CME after cataract surgery [20]. These findings underscore the importance of thorough preoperative assessment and patient counseling regarding the increased risk of CME in individuals with ERM.​ In patients with uveitis, the incidence of postoperative CME is notably higher. A study by Bélair et al. found that the incidence of CME at one month after cataract surgery was 12% in uveitic eyes compared to 4% in control eyes, and at three months, the incidence was 8% in uveitic eyes versus 0% in controls [21]. Given that our study excluded patients with uveitis and other inflammatory conditions, our CME incidence may reflect a lower-risk population compared to studies including these subgroups. This highlights the importance of meticulous perioperative management in patients with underlying inflammatory diseases, as their risk of CME remains significantly higher.

The type of IOL implanted has long been discussed in the context of CME pathogenesis. In our study, acrylic, silicone, and monoblock PMMA lenses were used according to availability, as standardized lens selection practices and technology were still evolving during the late 1990s [22]. No apparent correlation was observed between IOL material and CME development in our cohort. At the time, lens implantation techniques and available IOL types were limited, and hydrophobic acrylic IOLs - now recognized for their superior biocompatibility - were only beginning to be introduced. While our study was not powered to detect statistically significant differences between lens types, more recent evidence suggests that hydrophobic acrylic IOLs may induce less postoperative inflammation and consequently reduce the risk of CME compared to silicone or PMMA lenses. For example, modern comparative studies have shown that acrylic lenses are associated with lower anterior chamber flare and fewer inflammatory mediators, which may contribute to their favorable profile regarding CME risk [23]. Given the temporal context of our study, these findings should be interpreted with caution and viewed as a historical snapshot.

Recent literature underscores the significance of IOL material and design in the development of CME after phacoemulsification. A Cochrane review evaluates the outcomes of different IOL types in patients with uveitis undergoing cataract surgery. The analysis indicated that hydrophobic acrylic IOLs are associated with better postoperative outcomes, including a lower incidence of inflammation and CME, compared to other IOL materials. The review underscores the importance of IOL material in managing patients at higher risk for postoperative inflammation and CME [23]. Another comprehensive review discusses the incidence, risk factors, pathogenesis, and management of pseudophakic CME. It emphasizes that while modern surgical techniques have reduced the incidence of CME, it remains a significant cause of visual impairment after cataract surgery. The article highlights that the development of CME is multifactorial, involving surgical trauma, inflammation, and patient-specific factors. Regarding IOL characteristics, the review suggests that certain IOL materials and designs may influence the inflammatory response, potentially affecting the risk of CME [24]. These findings suggest that careful selection of IOL material can be a critical factor in minimizing the risk of CME following cataract surgery.

The evolution of IOL technology has significantly impacted postoperative outcomes, particularly in terms of lens location. In the early years of modern cataract surgery, both anterior chamber IOLs (ACIOL) and posterior chamber IOLs (PCIOL) were utilized depending on surgical circumstances [25-28]. However, our study, conducted in an era when PCIOLs were gaining popularity, primarily included patients who underwent uncomplicated phacoemulsification with PCIOL implantation. We observed no significant correlation between IOL position and the development of CME.

This finding is in line with the current standard of care, where PCIOLs have become the default choice due to their anatomical placement within the capsular bag, offering improved centration, reduced contact with corneal endothelium, and a lower risk of postoperative complications compared to ACIOLs [26]. Indeed, ACIOLs are now rarely used in routine cataract surgery, primarily reserved for cases with inadequate posterior capsular support [27]. Although recent advances in PCIOL technology, such as sutureless scleral fixation, have expanded the range of surgical options in complex cases, these techniques represent a distinct clinical context and were beyond the scope of our study, which focused on low-risk, uncomplicated cases [28].

This study has several limitations, primarily related to the era in which it was conducted. The relatively small sample size, limited availability of advanced imaging modalities (such as OCT), and restricted IOL options - mainly dictated by the surgical resources of the time - are notable constraints. Additionally, the gradual decrease in the number of eyes evaluated at later follow-up points, particularly by the sixth month, represents a limitation that may affect the generalizability and interpretation of long-term CME incidence in this cohort.

Despite these limitations, the study provides valuable insight into the early clinical outcomes of phacoemulsification and posterior chamber IOL implantation during a pivotal period in ophthalmic surgery. It reflects the surgical practices, decision-making processes, and diagnostic approaches of the late 1990s, making it a historically relevant dataset. In this context, the findings serve not only to assess postoperative CME incidence but also to document the evolution of cataract surgery techniques and diagnostic standards.

Over the past three decades, rapid advances in diagnostic technologies have fundamentally transformed the identification and management of complications such as CME. While FFA is limited to detecting vascular leakage, modern OCT devices can measure retinal thickness at sub-millimeter precision. Importantly, OCT allows for the detection of subclinical CME even in the absence of subjective visual complaints. The 11.1% CME rate identified in our study using FFA might have been higher if current imaging technologies had been available at the time. This discrepancy highlights how diagnostic tools shape our perception of disease and how historical data can be reinterpreted in light of technological progress. A comparative overview of the diagnostic, surgical, and material-related standards from the 1997-1998 period versus current practices is presented in Table 4, highlighting the key advancements that have influenced clinical outcomes and CME detection.

**Table 4:** Historical and current standards in cataract surgery: impact on CME diagnosis and outcomes FFA: fundus fluorescein angiography; OCT: optical coherence tomography; IOL: intraocular lens; PMMA: polymethylmethacrylate

Parameter	1997–1998 Data	2025 Standards	Clinical Implication	References
Diagnostic Method	FFA (invasive, analog)	OCT (non-invasive, high-resolution)	Subclinical cases can be detected earlier	[11,29]
CME Incidence	11.1% (angiographic)	~0.8% (clinically significant)	Increased diagnostic sensitivity and improved surgical outcomes	[1]
IOL Type	PMMA, silicone, acrylic (based on availability)	Hydrophobic acrylic (standard)	Less inflammation, better outcomes	[23, 30]
Surgical Approach	Large incision, Transition period: sutured/sutureless	Microincision, fully sutureless	Less trauma, faster healing	[28,31]
Imaging	Film-based, analog imaging	Digital, 3D cross-sectional analysis	Improved accuracy and speed in postoperative follow-up	[18,32]

These observations underscore how diagnostic capabilities shape clinical interpretation. While the absence of OCT in our cohort reflects the technological limitations of the time, it also highlights the evolving nature of clinical insight. Revisiting historical data with today’s diagnostic perspective not only deepens our understanding of past surgical outcomes but also reinforces the importance of adapting follow-up strategies in light of both technological and biological complexity.

This study presents the incidence of CME following uncomplicated cataract surgery in a patient cohort from the 1997-1998 period. Despite the limitations of diagnostic methods and surgical techniques used at the time, the observed incidence remains noteworthy when re-evaluated in light of current knowledge. Although modern surgical techniques, intraocular lens designs, and imaging modalities have significantly advanced, the literature indicates that CME continues to be reported. This suggests that CME is not solely related to technical factors but also influenced by biological mechanisms such as the inflammatory response. Our findings highlight that revisiting historical data through the lens of contemporary knowledge can enhance awareness around the diagnosis and management of postoperative complications.

## Conclusions

This study provides meaningful insight into the incidence and natural course of CME following uncomplicated phacoemulsification and posterior chamber IOL implantation during the late 1990s. Angiographic CME was identified in a notable proportion of patients, though most cases were asymptomatic and resolved without intervention. The higher incidence among older individuals reinforces age as a persistent risk factor, suggesting the importance of tailored postoperative monitoring in this population. The findings also illustrate that, even in the absence of advanced imaging technologies, clinically significant trends in CME incidence and resolution could be observed. Recognizing these patterns remains relevant today, as CME continues to be a common and multifactorial postoperative complication.

Ultimately, this study contributes to a broader understanding of CME's natural course and underscores the value of stratified risk assessment in cataract surgery follow-up care. These insights may support more personalized approaches to postoperative surveillance and patient counseling.

## References

[1] Iftikhar M, Dun C, Schein OD, Lum F, Woreta F (2023). Cystoid macular edema after cataract surgery in the United States: IRIS® registry (Intelligent Research in Sight) analysis. Ophthalmology.

[2] Milch FA, Yannuzzi LA (1987). Medical and surgical treatment of aphakic cystoid macular edema. Int Ophthalmol Clin.

[3] Grewal DS, Jaffe GJ (2017). Diagnosis of cystoid macular edema: imaging. https://link.springer.com/chapter/10.1007/978-3-319-39766-5_3.

[4] Ferro Desideri L, Arun K, Bernardi E, Sagurski N, Anguita R (2025). Incidence, pathogenesis, risk factors, and treatment of cystoid macula oedema following cataract surgery: a systematic review. Diagnostics (Basel).

[5] Buratto L, Brint S, Sacchi L (20241). Cataract Surgery: Introduction and Preparation. https://www.taylorfrancis.com/books/edit/10.1201/9781003522928/cataract-surgery-lucio-buratto-laura-sacchi-stephen-brint.

[6] Miyake K (1984). Indomethacin in the treatment of postoperative cystoid macular edema. Surv Ophthalmol.

[7] Niederer RL, Sharief L, Bar A, Lightman SL, Tomkins-Netzer O (2017). Predictors of long-term visual outcome in intermediate uveitis. Ophthalmology.

[8] Chu CJ, Johnston RL, Buscombe C, Sallam AB, Mohamed Q, Yang YC (2016). Risk factors and incidence of macular edema after cataract surgery: a database study of 81984 eyes. Ophthalmology.

[9] Wielders LH, Schouten JS, Aberle MR (2017). Treatment of cystoid macular edema after cataract surgery. J Cataract Refract Surg.

[10] Mangla R, Venkatesh R, Prabhu V (2023). Mid-phase pinpoint hyperfluorescent spots on fundus fluorescein angiography in acute central retinal artery occlusion - a novel imaging finding. Int J Retina Vitreous.

[11] Balhaddad AA, Alharamlah F, Albrahim HF, Ahmad S, Melo MA, Mokeem L, Gad MM (2025). Assessing diagnostic accuracy and monitoring of caries progression using optical coherence tomography (OCT): a systematic review. J Dent.

[12] Kumar S (2023). Fundus photography, fluorescein angiography, optical coherence tomography and electroretinography of preclinical animal models of ocular diseases. Ann Eye Sci.

[13] Arana LA, Pinto AT, Chader GJ (2012). Fluorescein angiography, optical coherence tomography, and histopathologic findings in a VEGF(165) animal model of retinal angiogenesis. Graefes Arch Clin Exp Ophthalmol.

[14] Fine HI, Packer M, Hoffman RS (2002). New phacoemulsification technologies. J Cataract Refract Surg.

[15] Boulter T, Bernhisel A, Mamalis C, Zaugg B, Barlow WR, Olson RJ, Pettey JH (2019). Phacoemulsification in review: optimization of cataract removal in an in vitro setting. Surv Ophthalmol.

[16] Starr MR, Cai L, Obeid A (2021). Risk factors for presence of cystoid macular edema following rhegmatogenous retinal detachment surgery. Curr Eye Res.

[17] Trichonas G, Kaiser PK (2014). Optical coherence tomography imaging of macular oedema. Br J Ophthalmol.

[18] Hosseini F, Asadi F, Rabiei R, Kiani F, Harari RE (2024). Applications of artificial intelligence in diagnosis of uncommon cystoid macular edema using optical coherence tomography imaging: A systematic review. Surv Ophthalmol.

[19] Padidam S, Skopis G, Lai MM (2022). Prevalence of cystoid macular edema after cataract surgery in eyes with previous macular surgery. Clin Ophthalmol.

[20] Chen YC, Chen SJ, Li AF, Huang YM (2022). Visual outcomes and incidence of pseudophakic cystoid macular oedema in eyes with cataract and idiopathic epiretinal membrane after two-step sequential surgery. Eye (Lond).

[21] Bélair ML, Kim SJ, Thorne JE, Dunn JP, Kedhar SR, Brown DM, Jabs DA (2009). Incidence of cystoid macular edema after cataract surgery in patients with and without uveitis using optical coherence tomography. Am J Ophthalmol.

[22] Apple DJ (2000). Influence of intraocular lens material and design on postoperative intracapsular cellular reactivity. Trans Am Ophthalmol Soc.

[23] Leung TG, Lindsley K, Kuo IC (2014). Types of intraocular lenses for cataract surgery in eyes with uveitis. Cochrane Database Syst Rev.

[24] De Maria M, Iannetta D, Cimino L, Coassin M, Fontana L (2020). Measuring anterior chamber inflammation after cataract surgery: a review of the literature focusing on the correlation with cystoid macular edema. Clin Ophthalmol.

[25] Yalçınkaya Çakır G, Altan Ç, Çakır İ (2024). Anterior chamber flare and choroidal vascular index as inflammatory markers after uncomplicated phacoemulsification surgery. Int Ophthalmol.

[26] Hannush SB (2024). Corneal considerations for noncapsular IOL fixation. Advanced IOL Fixation Techniques.

[27] Obasuyi OC, Tagar BO, Ewoigbe JO (20241). Short-term visual outcome following anterior chamber intraocular lens implantation—the experience of a rural teaching hospital. Niger J Ophthalmol.

[28] Sun H, Wang C, Wu H (2024). Recent advances and current challenges in suture and sutureless scleral fixation techniques for intraocular lens: a comprehensive review. Eye Vis (Lond).

[29] Mitne S, Paranhos Júnior A, Rodrigues AP (2003). Concordância entre tomografia de coerência óptica e angiofluoresceinografia no edema macular cistóide secundário a cirurgia de catarata [Agreement between optical coherence tomography and fundus fluorescein angiography in post-cataract surgery cystoid macular edema]. Arq Brasil Oftalmol.

[30] Luo C, Wang H, Chen X, Xu J, Yin H, Yao K (2022). Recent advances of intraocular lens materials and surface modification in cataract surgery. Front Bioeng Biotechnol.

[31] Dole K, Baheti N, Deshpande R, Kulkarni S, Shetty R, Deshpande M (2022). Comparative study of anatomical and functional recovery of eye along with patient satisfaction score after small-incision cataract surgery and phacoemulsification cataract surgery. Indian J Ophthalmol.

[32] Jorzik JJ, Bindewald A, Dithmar S, Holz FG (2005). Digital simultaneous fluorescein and indocyanine green angiography, autofluorescence, and red-free imaging with a solid-state laser-based confocal scanning laser ophthalmoscope. Retina.

